# NetiNeti: discovery of scientific names from text using machine learning methods

**DOI:** 10.1186/1471-2105-13-211

**Published:** 2012-08-22

**Authors:** Lakshmi Manohar Akella, Catherine N Norton, Holly Miller

**Affiliations:** 1 MBLWHOI Library, Marine Biological Laboratory, Woods Hole, MA, USA; 2 Present address: Sears Holdings Corporation, Hoffman Estates, IL 60179, USA

## Abstract

**Background:**

A scientific name for an organism can be associated with almost all biological data. Name identification is an important step in many text mining tasks aiming to extract useful information from biological, biomedical and biodiversity text sources. A scientific name acts as an important metadata element to link biological information.

**Results:**

We present NetiNeti (Name Extraction from Textual Information-Name Extraction for Taxonomic Indexing), a machine learning based approach for recognition of scientific names including the discovery of new species names from text that will also handle misspellings, OCR errors and other variations in names. The system generates candidate names using rules for scientific names and applies probabilistic machine learning methods to classify names based on structural features of candidate names and features derived from their contexts. NetiNeti can also disambiguate scientific names from other names using the contextual information. We evaluated NetiNeti on legacy biodiversity texts and biomedical literature (MEDLINE). NetiNeti performs better (precision = 98.9% and recall = 70.5%) compared to a popular dictionary based approach (precision = 97.5% and recall = 54.3%) on a 600-page biodiversity book that was manually marked by an annotator. On a small set of PubMed Central’s full text articles annotated with scientific names, the precision and recall values are 98.5% and 96.2% respectively. NetiNeti found more than 190,000 unique binomial and trinomial names in more than 1,880,000 PubMed records when used on the full MEDLINE database. NetiNeti also successfully identifies almost all of the new species names mentioned within web pages.

**Conclusions:**

We present NetiNeti, a machine learning based approach for identification and discovery of scientific names. The system implementing the approach can be accessed at http://namefinding.ubio.org.

## Background

There is a vast and ever growing amount of literature in biology, ecology, biomedicine, biodiversity, genomics and proteomics. The U.S National Library of Medicine’s MEDLINE [1] database is one such source with more than 18 million abstracts of journal articles in life sciences with focus in biomedicine. Major efforts to digitize legacy literature undertaken by consortiums like the Biodiversity Heritage Library (BHL) [2] generate vast amounts of text data from the Optical Character Recognition (OCR) of scanned literature. Extraction of knowledge from sources like MEDLINE can significantly speed up biomedical research by providing access to relevant information about diseases, genes, gene-protein, protein-protein interactions, model organisms and drugs. While gene/protein identifications and binary interactions have been the focus of biomedical text mining, more ambitious tasks like identifying complex nested structures are also being pursued currently [3].

Identification of species names and the normalization task of mapping them to identifiers in a database are considered essential sub-tasks for many text mining projects [4,5] like recognizing gene names [6-8] or extracting organism-specific information like life history, geographic distribution and predator–prey relationships from biodiversity and biomedical literature. A scientific name is a genus name or a species level name with genus followed by species or a name below the species level with genus, species and subspecies information. It can also be a higher order taxonomic name like family, order, etc. A scientific name is one of the named entities that can be connected with other entities like gene names, protein names, geographic locations, diseases, common names of organisms and names of people who first described the species. Recognition of named entities is frequently a first step in the process of performing more complex information extraction tasks like finding relations between the named entities or for question answering [9,10]. The name of an organism is one of the few identifying elements associated with almost all biological data [11]. A scientific name extraction system will be very useful in gathering all contexts in the form of sentences or paragraphs associated with organism names. These sentences and paragraphs can help enrich the existing content and add new content for projects like the Encyclopedia of Life (EOL), which aims to create a webpage for every single species on Earth [12]. Natural language processing and machine learning methods can be applied to extract fine-grained, atomic information that can be used to populate biological databases and repositories. The organism name serves as an important metadata element for linking information from various biological sources [13-16], so a species name identification system is an essential tool in information integration.

Most of the approaches in the literature addressing the problem of name finding from text sources primarily rely on dictionaries with a list of scientific and/or common names [4,14,17,18]. TaxonGrab [17] is a dictionary-based approach that uses a dictionary generated by combining dictionaries of English words and biomedical terms instead of a list of scientific names. Words that do not appear in this dictionary (inverse lexicon) and that follow simple rules for capitalization, abbreviations, variants and subspecies mentions used in scientific names are considered as organism names. Approaches that primarily rely on this kind of an inverse lexicon tend to have low precision as this can gather many false positives from misspelled English words, OCR errors and non-English words that pass through the rule filters. The precision of the system can also vary significantly from one text source to another depending on the number of words covered by the inverse lexicon. Hence such a system is also likely to perform very poorly on non-English texts.

TaxonFinder [14] is designed to find scientific names from text with the help of separate dictionaries for species and genus names. Though the approach is likely to have fewer false positives, the number of false negatives (the number of correct names missed) can be high as it cannot find anything that is not a genus and species combination from the dictionaries used in the approach. Such an approach cannot find misspelled names, names with OCR errors, new species names and other names not present in the dictionary. Such a system can also have false positives due to the presence of incorrect names, names that are spelled the same as some common English words and geo-location names (e.g. major, Atlanta).

The approach “Linnaeus” [4] uses dictionaries for scientific and common names to construct a DFA (Deterministic Finite Automaton) [19] to match species names. The system also tries to resolve acronyms for organisms (e.g. HIV, CMV) using the frequencies of most commonly used acronyms in MEDLINE calculated using Acromine [20]. Linnaeus only focuses on finding species names and currently does not deal with genera or other higher-order taxonomic units. Inherently being a dictionary based approach, Linnaeus also will have issues that were discussed above for approaches like TaxonFinder. There are also other dictionary-based approaches that identify species names based on the NCBI taxonomy [21,22]. FAT (Find All Taxon names) [18] is another tool that uses a combination of rules, dictionaries of scientific names and non-names along with input from users to find scientific names. Wang et al. [8,23,24] developed approaches to tag and disambiguate genes, proteins and protein-protein interaction with species names from the NCBI taxonomy, Uniprot [25] and manually created dictionaries using a rule based approach and/or with a machine learning based classifier. Their main objective was to disambiguate gene/protein or protein-protein mentions in text using species tags.

Here we focus on recognition/discovery of scientific names of organisms from various text sources. The problem of discovery of binomial and trinomial scientific names along with genera and higher taxonomic units can be quite complex. For example, biodiversity literature and legacy text sources like BHL (Biodiversity Heritage Library) contain many names with OCR errors, alternative names and misclassified names. Thousands of new species are discovered every year and many are reclassified. Some names are spelled the same as geo-locations or people names and therefore disambiguation of names is required. We have developed approaches and built tools that address all of the above.

NetiNeti is a solution for scientific name recognition/discovery. This approach enables finding scientific names in literature from various domains like biomedicine and biodiversity. It can discover new scientific names and also find names with OCR errors and variations. The system is based on probabilistic machine learning methods where a given string has a certain probability of being a scientific name or not being a scientific name depending on the name string itself and the context in which it appears. NetiNeti builds a machine learning classifier from both the structural features of a string and its contextual features. In the process of classifying a string, the approach can differentiate between common words like names of places or people from scientific names based on the context in which a name appears. For example, *Atlanta* is a scientific name in the sentence, “Atlanta is a genus of pelagic marine gastropod molluscs”. However, in the sentence, “The city Atlanta is in the state of Georgia”, *Atlanta* is a geographic location and not a genus name. NetiNeti correctly recognizes the word *Atlanta* as a scientific name in the first context and does not recognize it as a scientific name in the second context. Simple rules for capitalization and abbreviations in species names are applied as a pre-filtering step to generate candidate names. Candidates with common English words were also removed in the pre-filtering process. The candidate names along with their contexts are then classified using a supervised machine learning classifier. While the system can disambiguate and discover what scientific names of organisms are mentioned in a document, the approach is not about discovering documents that are about specific organisms based on their presence in the document.

We evaluated NetiNeti on legacy biodiversity texts (BHL books) and biomedical literature (MEDLINE). We compared results of NetiNeti and a dictionary based scientific name finder with the results of manual annotation of a BHL book. A comparison of some of the probabilistic machine learning algorithms on our annotated dataset for scientific name finding is presented. We also present the results of running NetiNeti on other biological text sources.

## Methods

### Pre-filtering and candidate generation

The input text is first tokenized using a tokenization scheme that breaks the characters in a stream of characters in natural language text into distinct meaningful units called tokens. We followed the conventions used by the Penn Treebank project [26] to tokenize text. Word trigrams, which are groups of three tokens along the token-sequence are then generated from the tokenized text and each trigram is then passed through a simple rule filter which checks if the tokens in the trigram have the right capitalization, abbreviations, etc. and checks if the trigram has no common English words. Each trigram that passes through the rule filter is then classified by a machine learning classifier as “scientific-name” or “not-a-scientific-name” using the structural and contextual features of the trigram. The trigram that was classified as a scientific name corresponds to a trinomial name, which is a name below the species level with genus, species and usually a subspecies. If a trigram fails to pass though the rule filter, the first two tokens (word bigram) of the trigram are then tested to see if they can become a candidate for a binomial name, with genus followed by a species mention. The classifier then classifies such candidate bigrams. Similarly, the first token of a failed bigram is analysed if it can become a candidate for a uninominal name (genus or higher order taxonomic unit), which gets classified accordingly if it is deemed as a candidate. NetiNeti also resolves abbreviated species names by noting that an abbreviation can be used for a species after a mention of its genus or an abbreviation can follow a mention of a full name (genus-species combination) or an abbreviated name for a species can be used after a mention of another species name from the same genus.

### Machine learning based classification

We applied probabilistic machine learning algorithms like Naïve Bayes and Maximum Entropy to classify candidate names. The objective is to estimate the probability of a label (whether a name is scientific or not) given a candidate string along with its contextual information. Naïve Bayes and Maximum Entropy classifiers learn or estimate the probabilities from a training set.

(1) $$P\left(c_{i}|s_{j}\right)=\frac{P\left(c_{i},s_{j}\right)}{P\left(s_{j}\right)}=\frac{P(s_{j}|c_{i}P\left(c_{i}\right)}{P\left(s_{j}\right)}\approx \frac{1}{P\left(s_{j}\right)}P\left(c_{i}\right)\prod_{k=1}^{K}P\left(f_{k}|c_{i}\right)$$

(2) $$label=\arg\max_{c_{i}\in C}[\log\left(P\left(c_{i}\right)\right)+\sum_{k=1}^{K}\log\left(P\left(f_{k}|c_{i}\right)\right)]$$

We are primarily interested in the conditional probability of a class label, $c_{i}\in C=\left\{'yes','no'\right\}$ given an input string and its contexts *s*_*j*_ as in Eq.1. The ‘yes’ and ‘*no’* labels correspond to whether a string is a scientific name or not. Once we get these conditional probabilities, we simply choose the label with the highest probability for a given string. The Naïve Bayes classifier [27-29] as seen in Eq.1. actually models the joint probability $P\left(c,s\right)$ of a class *c* and a string *s* and makes an assumption that all the features $f_{1},f_{2},\ldots f_{K}$for the string and its contexts given the class label are independent as in Eq.1 This independence assumption is strong, but it helps to easily estimate the probability $P\left(s_{j}|c_{i}\right)$, of a string *s*_*j*_ given the class label *c*_*i*_ from a training set of labelled examples. Even with this independence assumption, the Naïve Bayes classifier performs surprisingly well in many document classification tasks [27,29]. $P\left(f_{k}|c_{i}\right)$ can be estimated from the number of training examples having the feature value *f*_*k*_, and the number of examples with class label *c*_*i*_ and also having the feature value *f*_*k*_ We can then get the class label for a string (along with its contexts) from Eq.2 with probabilities taken in the log scale.

The Naïve Bayes approach is called *generative* as it is based on a model of the joint distribution *P*(*c*, *s*). The maximum entropy classifier, also known as a logistic regression classifier, is called a *discriminative* approach as it is based on the model of the conditional distribution *P*(*c*|*s*) Maximum entropy is widely used for many natural language processing tasks like text segmentation [30], parts-of-speech tagging [31], language modelling [32], text classification [33] and Named Entity Recognition (NER) [9,10]. The principle behind the maximum entropy approach is to model all that is known and assume nothing about what is unknown [34]. Given a collection of facts (in the form of a training set), the approach chooses a model that is consistent with all facts with a distribution that is as uniform as possible i.e., the distribution that allocates its probability as evenly as possible obeying all the constraints derived from the training set. The conditional probability of a label $c_{i}$ given the string context $s_{j}$ takes the following exponential form [35] in Eq.3.

(3) $$P(c_{i}|s_{j})=\frac{1}{Z\left(s_{j}\right)}\exp\left[\sum_{m=1}^{M}\lambda_{m}g_{m}\left(c_{i},s_{j}\right)\right]$$

Where each $g_{m}\left(c_{i},s_{j}\right)$ is a binary valued feature function defined on the class label and the string context, $\lambda_{m}$s are the weights to be learned from the training set for the feature functions and $Z\left(s_{j}\right)=\sum_{c_{i}}\exp(\sum_{m=1}^{M}\lambda_{m}g_{m}\left(c_{i},s_{j}\right))$ is a normalizing factor that ensures that $\sum_{c_{i}}P\left(c_{i}|s_{j}\right)=1$. The parameters $\lambda_{m}$ are estimated via hill climbing approaches like Improved Iterative Scaling (IIS) [35] or Generalized Iterative Scaling (GIS) [36]. Limited-Memory Variable Metric optimization methods like L-BFGS [37] have been found to be effective for Maximum Entropy parameter estimation [38]. In our scientific name recognition task, we have applied and compared the IIS, GIS and L-BFGS methods for parameter estimation on a corpus that was manually annotated with scientific names. For both Naïve Bayes and the Maximum Entropy classifiers, we used the Python [39] implementations in the NLTK [40] package. MEGAM [41] optimization package was used for L-BFGS optimization.

### Training set generation

An initial set of about 5,000 names was used as a positive example set. Candidate strings from unigram, bigram and trigrams of a tokenized BHL book [42], which does not contain any scientific names, was used as an initial negative example set. An initial maximum entropy classifier was trained with the initial training set using only the structural features of strings. A set of MEDLINE abstracts, a small portion of content from EOL [12] and biodiversity texts from BHL were segmented into sentences using the sentence tokenizer in NLTK, pre-filtering and candidate generation steps were performed for each sentence, and the initial classifier was used to get scientific names that were identified with high confidence. The scientific names along with the sentences in which they occur together form the positive example set. Features were derived from the scientific names and a neighborhood of word contexts appearing around the scientific names in the sentences. We tokenized a geography book from the Internet archive [42] and the strings derived from word unigrams, bigrams, and trigrams in the tokenized text of the book form the negative example set. About 10,000 positive examples with contextual information, another 10,000 examples from scientific names without contextual information were used as the positive example set. Abbreviated names from these examples were also added to the positive example set. A total of about 40,000 positive examples together with another set of about 43,000 negative examples were used to generate a training set of 83,000 examples for the two class labels. Features used include the last three, last two and the last characters along with the first and second characters of the unigram, bigram, and trigram candidates. Binary features like whether the last, second last, and third last characters are present in different partitions of the set, *’a’,’e’,’i’,’o’,’u’,’s’,’m’* were also used. Presence or absence of a particular word in unigram, bigram, and the trigram candidates in a dictionary of genus and species combinations were also part of the binary features. When a word token is part of the dictionary of names it contributes to the conditional probability of the candidate name given the structural and contextual features. Numerical features like the number of vowels in various parts of the candidate names were also used. For contextual features, words appearing in the neighborhood of candidate names and their parts-of-speech tags were used.

## Results and discussion

### Evaluation sets

NetiNeti focuses on discovering/identifying scientific names of organisms including names with spelling and OCR errors from text sources across domains like biodiversity and biomedicine. We present the results of running NetiNeti on three different text sources.

BHL is a rich source of biodiversity data with over 80,000 volumes corresponding to over 30 million scanned pages converted to text. A gold-standard biodiversity corpus marked with scientific names by an annotator was created, as there are no previously reported annotated corpora for biodiversity information. Also, the evaluation sets that were previously reported were not specifically annotated for scientific names of species along with errors and variations. All the scientific names, including names with OCR errors, occurring in a 600 page BHL book “American Seashells” [43] were extracted manually by the annotator. We used NetiNeti to identify all names in this book and compared our results to the list of names that were manually extracted. We also compared our results with the results of the dictionary-based TaxonFinder [44] and the FAT tool integrated into the GoldenGATE editor [45] for finding scientific names The comparison results have been summarized in Table 1. We also ran NetiNeti on MEDLINE, which contains over 18 million bibliographic records from journal articles in life sciences with a concentration on biomedicine. We present the results of running two of the best performing algorithms against the MEDLINE database summarized in Table 2. We also evaluated NetiNeti on a small subset of 136 tagged PubMed Central’s (PMC) [46] open access full-text articles. These 136 articles were selected from the evaluation set used by Linnaeus species identification system [4] with only scientific name tags, as their full PMC evaluation set consists of articles also tagged with common names.

**Table 1:** Precision and recall values for NetiNeti, TaxonFinder and FAT on the american seashell book

**APPROACH**	**PRECISION**	**RECALL**	**F-SCORE**
NetiNeti	0.989	0.705	0.8231
TaxonFinder	0.975	0.543	0.6975
FAT	0.840	0.402	0.5437

**Table 2:** Results of running NetiNeti with Naïve Bayes and MaxEnt (GIS) on MEDLINE

**Algorithm**	**Unique**	**Binomial and Trinomials**	**PMIDs covered**
Naïve Bayes	227796	193596	1883750
MaxEnt	214352	188606	1551176

### Comparison of machine learning classifiers

**Figure 1:**
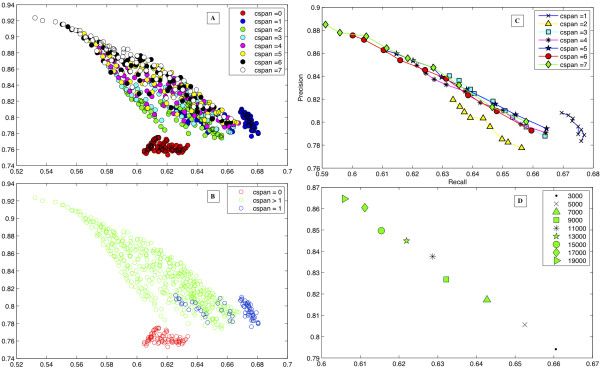
**Precision and recall plots for various parameter values and settings on the American Seashells test data.A**, Plot of precision and recall (P-R) values with different training set sizes and different neighborhoods for contextual features indicated with cspan values corresponding to the number of contextual features from 0 to 7. **B**, This plot has precision and recall values with cspan = 0 (no contextual information), cspan =1 and cspan > 1. **C**, P-R plots with increasing training set of positive examples and different context spans (cspan = 1 to 7). **D**, Summarization of the results in C for cspan = 5 corresponding to 5 contextual features on either side of the candidate name. The stars in Figure 1**C**corresponding to cspan = 5 were all summarized in Figure 1**D**with different symbols. The star is Figure 1**D** is 4^th^ star from the top in Figure 1**C**.

We performed a series of training experiments with the Naïve Bayes classifier using different neighbourhoods for contextual features, different sizes of positive and negative training examples and evaluated the resulting classifiers with the precision and recall measures on the “American Seashells” book [43] using the manually extracted set of names from it. Precision is the fraction of the retrieved names that are relevant scientific names and recall is the fraction of scientific names retrieved from all the scientific names in a document. “cspan” in Figure 1 indicates the number of contextual features. When no contextual features were used, increasing the number of training examples did not yield any significant improvements in precision or recall as in Figure 1A indicated by the red circles which all clustered together. Figure 1B illustrates this more clearly, where all the red circles are close to each other in the P-R space. The blue circles are the result of using classifiers with a single contextual feature on either side of the candidate name. We can see that all the classifiers corresponding to the blue circles perform better than any of the classifiers corresponding to the red circles that did not use any contextual information during the training phase. All the circles colored other than red in Figures 1A and 1B represent the precision and recall values of classifiers trained with one or more contextual features on either side of the candidate names.

Figure 1C illustrates the effect of increasing number of contextual features and increasing the number of positive examples in the training set. For example, the blue stars in Figure 1C correspond to using five contextual features on either side of the candidate name with increasing positive example size during training. This was more clearly represented in Figure 1D, where we used five contextual features (cspan = 5) on either side of the candidate name for each classifier with increasing sizes of positive example sets form 3,000 to 19,000 in increments of 2,000 for training. It can be seen from Figure 1D that increasing the positive example set contributed to the better precision of the corresponding classifier with a slightly lower value for recall.

In our subsequent experiments we compared the precision and recall values of Naïve Bayes and Maximum Entropy classification algorithms with various parameter estimation methods like GIS, IIS, and L-BFGS on the manually annotated *American Seashell* book. We also compared the Decision Tree Learning algorithm [28,47] implemented in the NLTK toolkit. For the comparison of the algorithms, we used a context span of 1 corresponding to features derived from a word on either side of the candidate name for which the recall was higher than the other configurations with a good precision (> 0.8). Comparison of the algorithms was performed both with and without the use of a stop-list of English words used as part of the pre-filtering process as described in Methods. The results are summarized in Table 3. The Naive Bayes algorithm has the highest F-score (harmonic mean of precision and recall values) compared to other algorithms for this dataset when applied with and without a stop-list during pre-filtering. All the algorithms with the exception of the Decision Tree learning algorithm performed well with a better precision when a stop-list was used, although it did not have much impact on the recall values. Having a stop-list eliminates English words or other common words to generate a cleaner set of candidate names. However, the results from Decision Tree learning algorithm, which is an implementation of the C4.5 algorithm [47], are not significantly improved through use of the stop-list. If we have more labelled datasets for scientific name recognition, it would be interesting to see how well the learned decision tree performs on them. The Maximum Entropy algorithm with the limited memory variant of the BFGS algorithm also performs well with a high precision of 0.97 with a stop-list and 0.88 without the stop-list, but the recall values are relatively lower. However, with the GIS estimation, the Maximum Entropy approach has the second best F-score of 0.7455 after the Naïve Bayes algorithm as shown in Table 3.

**Table 3:** Precision and recall values for naïve bayes, maximum entropy (iis, gis, l-bfgs) and decision tree learning algorithms on the american seashells book

**ALGORITHM**	**STOPLIST**	**PRECISION**	**RECALL**	**F-SCORE**
Naïve Bayes	Yes	0.9487	0.6897	0.7987
Naïve Bayes	No	0.7901	0.6877	0.7353
MaxEnt (IIS)	Yes	0.9563	0.5951	0.7336
MaxEnt (IIS)	No	0.8175	0.5933	0.6875
MaxEnt (GIS)	Yes	0.9541	0.6118	0.7455
MaxEnt (GIS)	No	0.8151	0.6108	0.6983
MaxEnt (L-BFGS)	Yes	0.9707	0.5481	0.7006
MaxEnt (L-BFGS)	No	0.8883	0.5410	0.6724
Decision Tree	Yes	0.9820	0.5969	0.7424
Decision Tree	No	0.9793	0.5882	0.7349

### Results on biodiversity text with errors

**Figure 2:**
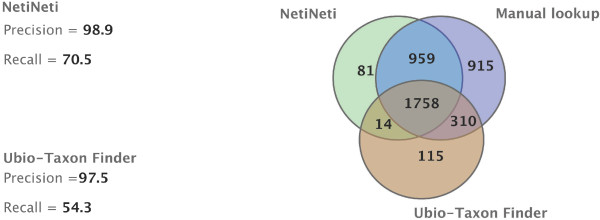
**Comparison between NetiNeti and TaxonFinder on *American Seashells *Book in BHL.**

Figure 2 summarizes the results of running the NetiNeti with Naïve Bayes algorithm on the annotated corpus (“*American Seashell*” book). We also compare our results with those of TaxonFinder. It can be seen that NetiNeti performs better both in terms of precision and recall. We further analysed the 81 names that did not match the manual lookup from NetiNeti and 115 names from TaxonFinder and noticed that among the 81 names, about 22 names were true false positives like geographic locations, common names and author names. The remaining 59 names were either a part of a scientific name, a different variant of a string that the system found from the one that was annotated, etc. Among the 115 names missed by TaxonFinder, about 40 names were true false positives and the rest of the names again were only part of a name or a different variant of a scientific name. The 14 names that are present in NetiNeti and TaxonFinder but not in the manual list were mostly parts of scientific names identified by both approaches and some common true false positives.

When calculating the precision and recall reported in Figure 2, we have taken into account only the true false positives. We can see that the recall for TaxonFinder is significantly lower compared to NetiNeti, while the precisions are comparable. For a dictionary-based approach like TaxonFinder, it is less likely to have many false positives as it only retrieves what is already present in a known set of names in the dictionary and so can have higher precision, but the recall can be very low as we have seen in the results summarised in Figure 2, the number of false negatives (the number of correct names missed) can be high as it cannot find anything that is not a genus and species combination from the dictionaries used. Such an approach also cannot handle misspelled names, names with OCR errors, new species names, or other names not present in the dictionary. NetiNeti on the other hand will handle these well and it is a name discovery tool. A comparison of NetiNeti, TaxonFinder and FAT tool for the BHL book is presented in Table 1. The FAT approach has lower precision and recall values compared to NetiNeti and TaxonFinder approaches for this corpus. The names marked up by the FAT tool were compared with the manual mark up. 869 of the names identified by FAT did not match with the manually marked up set of names. Most of these unmatched names are species epithets with authorship information. We further analyzed a random sample of 100 names out of these 869 names and examined genus information interpreted by the tool in the marked up tags. 32 of the 100 mismatched names have correctly interpreted genus names and the remaining are all true false positives with incorrect genus tags. We estimated that 278 of these 869 are correct identifications and the adjusted precision and recall values for the FAT approach were summarized in Table 1. For many of the true false positives, the FAT tool tags the species epithet, but does not seem to recognize the genus name immediately preceding the species name.

### Results on new species web pages

We have also conducted several small experiments on web pages with information about newly discovered species along with their scientific names. NetiNeti successfully discovers almost all the new species from the descriptions while the dictionary based TaxonFinder finds in most cases either only the genus or does not recognize the new name at all. The results were summarized in Table 4. The double starred names are those that were detected by NetiNeti and not detected by TaxonFinder. A few uninominal names that were not detected by NetiNeti but identified by TaxonFinder are displayed with a single star in the table. In this set, it can be seen that NetiNeti has only one false positive (indicated by ‘FP’) and was able to discover almost all of the new species’ mentions in web pages with new species. The name “Stephania” in the first entry in Table 4 corresponding to TaxonFinder is a false positive as the name in the context refers to a photographer not the genus “Stephania”.

**Table 4:** Comparison of NetiNeti and TaxonFinder on web pages with new species descriptions

**URL**	**NetiNeti**	**TaxonFinder**
http://www.livescience.com/environment/ top-10-new-species-1.html	Desmoxytes purpurosea **	Desmoxytes
	Electrolux addisoni **	Gryposaurus
	Gryposaurus monumentensis **	Megaceras
	Malo kingi **	
	Megaceras briansaltini **	Narkidae
	Narkidae	Oxyuranus temporalis
	Oxyuranus temporalis	Philautus maia*
	Philautus	Stephania-FP
	Styloctenium mindorensis	Styloctenium mindorensis
	Tecticornia bibenda **	Tecticornia
	Xerocomus silwoodensis	Xerocomus silwoodensis
http://news.mongabay.com/2010/041 9-hance_ microbes.html		
	Ceratium longipes	Ceratium longipes
	Culexiregiloricus trichiscalida **	Chlamydophrys*
	Lebbeus clarehanna **	Lebbeus
	Valdiviella insignis	Valdiviella insignis
http://species.asu.edu/2009_species05	S. ysbryda **	Selenochlamys
	Selenochlamys ysbryda **	Stylommatophora
	Trigonochlamydidae	Testacella *
		Trigonochlamydidae
http://species.asu.edu/2010_species09	G. carapo	G. carapo
	Gymnotus carapo	Gymnotidae
	Gymnotidae	Gymnotiformes*
	Gymnotus	Gymnotus
	Gymnotus omarorum **	Gymnotus carapo
http://species.asu.edu/2010_species03	Dioscorea orangeana	Dioscorea orangeana
	Dioscorea sambiranensis	Dioscorea sambiranensis
	Dioscoreaceae	Dioscoreaceae
http://species.asu.edu/2010_species02	Acrocirridae	Acrocirridae
	Swima bombiviridis **	Bombus
		Viridis-FP
http://species.asu.edu/2010_species01	Nepenthes attenboroughii **	Nepenthaceae
	Nepenthaceae	None-FP
http://species.asu.edu/2009_species06	Diplommatinidae	Diplommatinidae
	O. vermiculum **	None-FP
	Opisthostoma vermiculum **	Opisthostoma
http://species.asu.edu/2010_species06	Nephila	Nephila
	Nephila komaci **	Nephila turneri
	Nephila turneri	
	Nephilidae **	
	Habitus-FP	

### Results on PMC full text and MEDLINE

**Figure 3:**
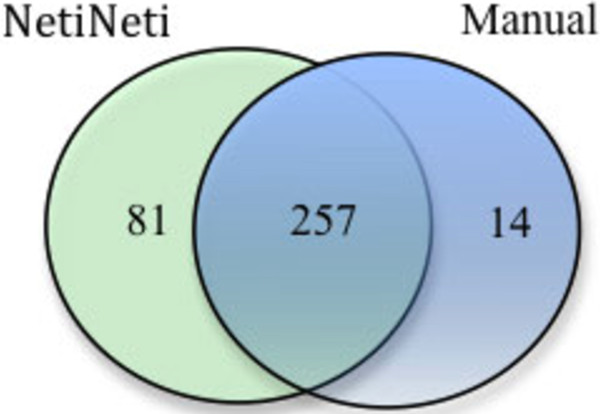
Comparison of results of netiNeti with 136 PMC full Text open access articles.

The results of running NetiNeti with Naïve Bayes algorithm for classification on 136 PMC full text articles are summarized in Figure 3. Here we chose a subset of the articles that were specifically tagged with scientific names from the set of articles tagged with both common names and species names as an evaluation set in Linnaeus system. Among the 81 names that did not match with the manual annotation, 76 names are scientific names with misspellings mostly in one or two characters and names that were missed by the annotators. Only 5 names were true false positives that do not correspond to any scientific names. So the precision and recall for NetiNeti on this data set were 0.985 and 0.962 respectively. The Linnaeus system deals with species level names including common names, so we cannot make a direct comparison with our system.

We also analysed the results of running NetiNeti on the whole of MEDLINE with Naïve Bayes and Maximum Entropy (GIS) classifiers, which were the top two algorithms in terms of F-scores in Table 2. The results were summarized in Table 2. NetiNeti with the Naïve Bayes algorithm found 193,596 unique binomial and trinomial names while the Maximum Entropy algorithm found 188,606 names. That is more than 3 times the number of species found by the dictionary-based Linnaeus system even though we focus only on scientific names. In the names extracted from MEDLINE, the errors include disease names like *Enterohepatitis*, terms like *Amputatio interilio-abdominalis* which was extracted from title of a PubMed article in Russian, chemical names like *Aminoanthracene*. Some of the errors in biodiversity text include terms like *Operculum corneous*, words associated with some geographic locations like *Panaina*. Biological terms and certain words associated with geographic locations can be the kind of errors common to both the corpora. Also, named entities with Latin-like endings can be incorrectly identified as scientific names of organisms by the system especially when there is little or no contextual information.

The system is highly scalable and we ran name finding on the recent update of MEDLINE with over 18 million abstracts in under 9 hours on a 2.8 Ghz intel core i7 based machine running Mac OX 10.6 using 6 cores.

As NetiNeti also extracts names with errors and variations, a need to map the names to known identifiers in a master list of names or a database arises. We are working on highly efficient methods based on suffix-trees to do such a mapping.

### Availability and requirements

The software system implementing NetiNeti can be accessed at http://namefinding.ubio.org. Currently a Naïve Bayes classifier is applied by default for name finding. The *American Seashell* book and a list of PubMed Central ids used for evaluation of NetiNeti can be found at http://ubio.org/netinetifiles

## Conclusions

In this article, we presented an approach for recognizing/discovering scientific names along with spelling errors and variations from various text sources in domains like biodiversity and biomedicine. We present NetiNeti as a solution to name discovery that uses machine learning techniques to classify candidate names generated by applying rules and pre-filtering methods on text. NetiNeti is highly scalable and configurable.

Whether to know the number of scientific names covered in a text, to extract all the sentences/paragraphs associated with scientific names or to tag mentions of genes, protein or other entities with scientific names or whether to incorporate species names as meta data elements for search, etc. or for taxonomic indexing, an identification and discovery tool like NetiNeti is very useful.

## Authors’ contributions

LMA designed, developed the appraoch and implemented the system, performed the experiments described and is a major contributor in the preparation of this manuscript. HJM supervised the project and provided support for manuscript preparation and contributed to the manuscript. CNN reviewed the draft and provided support for the project. All authors read and approved the final manuscript.
